# Dynamic evolution of left ventricular strain and microvascular perfusion assessed by speckle tracking echocardiography and myocardial contrast echocardiography in diabetic rats: Effect of dapagliflozin

**DOI:** 10.3389/fcvm.2023.1109946

**Published:** 2023-02-23

**Authors:** Juan Liu, Yixuan Wang, Jun Zhang, Xin Li, Lin Tan, Haiyun Huang, Yang Dai, Yongning Shang, Ying Shen

**Affiliations:** ^1^Department of Ultrasound, Southwest Hospital, Army Medical University (Third Military Medical University), Chongqing, China; ^2^Department of Cardiovascular Medicine, Rui Jin Hospital, Shanghai Jiaotong University School of Medicine, Shanghai, China

**Keywords:** early diabetes mellitus, microvascular strain, microvascular perfusion, speckle tracking echocardiography, myocardial contrast echocardiography, dapagliflozin

## Abstract

**Background:**

This experimental study aimed to determine the dynamic changes in myocardial strain and microvascular perfusion in diabetic rats by comprehensive echocardiography while evaluating the effect of dapagliflozin (DAPA).

**Materials and methods:**

Male Sprague–Dawley rats (*n* = 128) were randomly divided into four groups based on the presence or absence of a high-fat diet and streptozotocin-induced diabetes with or without DAPA treatment (*n* = 32/group). Serial conventional ultrasound, two-dimensional speckle tracking echocardiography (2D-STE) and myocardial contrast echocardiography (MCE) were performed at 2, 4, 6, and 8 weeks, and left ventricular global longitudinal strain (GLS), myocardial blood flow velocity (MBFV), myocardial blood flow (MBF), and myocardial blood volume (MBV) were determined. All animals were sacrificed immediately after the last echo measurement for histopathological assessment.

**Results:**

Despite similar conventional Doppler-echo indexes among the groups at 2, 4, 6, and 8 weeks (*p* > 0.05), left ventricular GLS, MBFV, MBF, and MBV were decreased at 8 weeks in diabetic rats (*p* < 0.05) as detected by both 2D-STE and MCE. These indexes were significantly improved at 6 and 8 weeks after treatment with DAPA for diabetic rats (*p* < 0.05), reaching similar values observed in non-diabetic controls. DAPA treatment was associated with increased myocardial vacuolization and microvessel density and reduced interstitial fibrosis in diabetic rats.

**Conclusions:**

Combined 2D-STE and MCE is sensitive for detecting left ventricular deformity and impaired microvascular perfusion in prediabetes and the early stage of diabetes mellitus. DAPA exerts a beneficial effect on protecting myocardial perfusion in diabetic rats.

## 1. Introduction

It is well recognized that chronic hyperglycemia reduces the self-protective ability of vascular endothelial cells, impairs microvascular perfusion, and decreases cardiac systolic and diastolic function, ultimately leading to diabetic cardiomyopathy. Cardiac dysfunction in the absence of coronary artery disease, hypertension, and valvular disease is called diabetic cardiomyopathy, which is one of the most common complications of diabetes mellitus (DM). The main pathological changes of diabetic cardiomyopathy includes myocardial interstitial fibrosis, myocardial stiffness and microvascular rarefaction, eventually leading to diastolic and systolic dysfunction. Timely intervention of diabetic cardiomyopathy is very challenging (1), therefore, the detection of alterations in myocardial and microvascular function in the process from prediabetes to the early stage of diabetes mellitus (earlyDM) plays a vital role in the prevention of serious complications (2). Although conventional ultrasound techniques, such as M-mode, two-dimensional echocardiography, coupled with color Doppler flow and tissue Doppler imaging, are routinely used for evaluating cardiac structure and function, two-dimensional speckle tracking echocardiography (2D-STE) provides quantitative assessment of local and global left ventricular mechanical changes (3), and myocardial contrast echocardiography (MCE) serves as a tool to determine the total amount and speed of myocardial microvascular blood perfusion (4). However, data are lacking concerning the role of these techniques used alone or in combination for evaluating dynamic changes in myocardial function and microvascular perfusion, especially for patients with diabetes mellitus.

Several randomized trials and observational studies have shown that sodium-glucose cotransporter 2 (SGLT2) inhibitors significantly reduce the risk of cardiovascular adverse events in diabetic patients (5–7). Further studies indicated that these agents could also provide beneficial cardiovascular effects even for non-diabetic patients, suggesting that myocardial protection with SGLT2 inhibitors may occur through a glucose-independent pathway (8–10). Nevertheless, further in-depth studies are warranted to clarify the mechanism of the protective effect of SGLT2 inhibitors on the myocardium.

Since changes in myocardial function and microvascular perfusion in prediabetes and earlyDM have not yet been well studied and whether early intervention with SGLT2 inhibitors can effectively prevent myocardial damage is not clear, in this experimental study, we used conventional ultrasound techniques, 2D-STE and MCE, to evaluate the dynamic evolution of myocardial function and microvascular perfusion and to explore the potential beneficial effect of the SGLT2 inhibitor dapagliflozin (DAPA) in a diabetic rat model.

## 2. Materials and methods

### 2.1. Animal model and groups

Male Sprague–Dawley rats (120–150 g) aged 5 weeks were obtained from the Laboratory Animal Center of the Third Military Medical University and kept in a constant temperature and humidity environment under a 12-h light/dark cycle with free access to food and water. The experimental protocol was approved by the Laboratory Animal Welfare and Ethics Committee of the Third Military Medical University (No. AMUWEC20224071).

**Figure 1:**
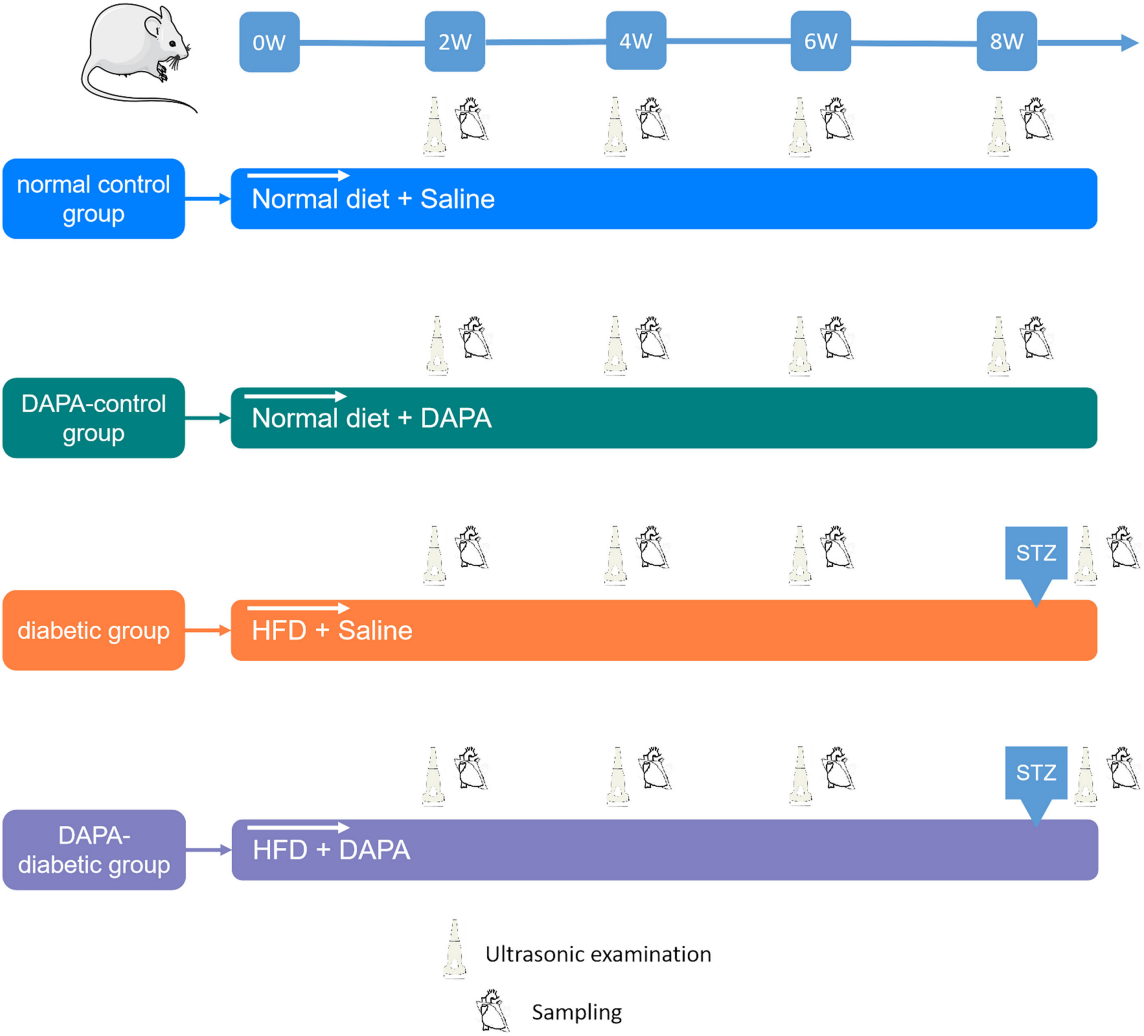
Study design. For each group, echo-Doppler, 2D-STE, and MCE were performed every 2 weeks. The myocardium was collected at sacrifice for histopathological analysis.

A total of 128 animals were randomly divided into four groups (*n* = 32 in each group): the normal control group included rats with 8 weeks of standard diet plus saline followed by citrate buffer; the DAPA-control group consisted of rats with 8 weeks of standard diet plus oral administration of DAPA followed by citrate buffer; the diabetic group comprised rats with 8 weeks of high-fat diet (HFD) plus saline followed by intraperitoneal injection of streptozotocin (STZ), and the DAPA-diabetic group comprised rats with 8 weeks of HFD plus oral administration of DAPA followed by intraperitoneal injection of STZ. In brief, a HFD consists of 79.85% general animal feed, 15% fat (oil), 5% custard powder, and 0.15% cholesterol (Laboratory Animal Center of the Third Military Medical University). DAPA (AstraZeneca) was given orally at a dose of 1 mg/kg/d for 8 weeks. STZ (Sigma–Aldrich, St. Louis, MO) was injected at a dose of 30 mg/kg under fasting conditions, and diabetes was identified when the fasting blood glucose level was >11.1 mmol/L at day 5 (Figure 1).

### 2.2. Conventional Doppler echocardiography

Ultrasound scanning of rats was performed under anesthesia with 3% isoflurane and oxygen with a heart rate maintained at 300~400 beats/min. Animals were placed on a prewarmed rodent platform, and body temperature was maintained at approximately 37°C. The anterior chest hair was then cleared.

An ultrahigh resolution animal imaging system (VEVO 2100, VisualSonics, Toronto, Canada) with a linear array probe (MS400, a frequency of 24 MHz) was used to carry out conventional transthoracic echocardiography. M-mode images were recorded at the level of the papillary muscles in the best parasternal long axis view. Left ventricular end-diastolic (EDD) and end-systolic diameters (ESD) and end-diastolic posterior wall thickness (PWd) and interventricular septum thickness in diastole (IVSd) were measured, and fractional shortening (FS) and left ventricular mass index (LVMI) were calculated. Left ventricular end-diastolic (EDV) and end-systolic volumes (ESV) and ejection fraction (EF) were determined by two-dimensional echocardiography. An average of three consecutive cycles was used.

Spectral Doppler in the best 4-chamber view with the sampling site positioned at the level of the mitral valve was used to measure peak flow velocity (E), isovolumic systolic time (ICT), isovolumic diastolic time (IRT), and LV ejection time (ET). Tissue Doppler imaging (TDI) was applied to determine myocardial motion velocity (e') at the junction between the septum and mitral annulus. The ratio of E to e' (E/e') and the Tei index, (ICT + IRT)/ET, were calculated (11).

### 2.3. Two-dimensional speckle tracking echocardiography

Parasternal long- and short-axis images were acquired for at least 3 cardiac cycles with optimal visualization of the LV myocardium. 2D-STE analysis was performed offline using TOMTEC software (TOMTEC Image System GmbH, Germany). Both endocardial and epicardial boundaries of the left ventricular myocardium were manually traced. This software can automatically track the movement of the entire myocardium. All tracked contours were checked, and, when poorly tracked, the data were excluded. Left ventricular global longitudinal strain (GLS), global circumferential strain (GCS), and global radial strain (GRS) were then derived. All values were averaged from 3 cardiac cycles.

### 2.4. Myocardial contrast echocardiography

Real-time MCE was performed using a Philips ultrasound diagnostic system (EPIC 7, Philips Medical Systems, Andover, MA, USA) with a linear array probe (eL18-4, 20 MHz). In brief, a commercially available contrast agent (SonoVue, Bracco, Italy) was diluted twice by adding 5 ml of normal saline, and a 24-gauge cannula was placed into the tail vein with continuous infusion (0.3–0.4 ml/min) (12). All images were optimized for each animal, including penetration depth 2 cm, near field focused on the middle of the left ventricle, gains adjusted without signal intensity of the myocardium, maximal dynamic range (60 dB), and mechanical index 0.06. When contrast agent was filled in the myocardium, left ventricular parasternal long axis images were stored for at least 20 cardiac cycles. The “flash (high energy pulse, mechanical index >1.0)” function was immediately triggered to destroy all myocardial microbubbles and then automatically switched to a low-energy real-time contrast state.

All video recordings were analyzed offline using a QLAB (version 6.0, Philips Healthcare) workstation. Regions of interest (ROIs) were manually placed on the middle segment of the anterior myocardium with an area of approximately 1.5 mm^2^, and each frame was manually checked to avoid partial volume effects from the right and left ventricular cavities. The first frame image after “flash” was set as the background frame, and then the time-signal intensity curve was fitted to an exponential function (13):


$$\begin{array}{l} Y=A\left(1-e^{-\beta t}\right)+C, \end{array}$$

where A is the peak intensity in the plateau phase, reflecting myocardial blood volume (MBV), β is the rising slope of signal intensity, reflecting myocardial blood flow velocity (MBFV), and A^*^β equals myocardial blood flow (MBF) (14).

### 2.5. Histopathological examination

After MCE, all animals were anesthetized in an unconscious state, then euthanized with thoracotomy and heart removal. The heart was fixed in 4% neutral buffered formalin and embedded in paraffin, then serially sectioned along the long axis of the left ventricular papillary muscle (4 μm thick), which was similar to the MCE plane. Hematoxylin and eosin (H&E) staining was performed to assess cardiac histomorphological changes. Masson's trichrome staining was performed to visualize the status of cardiac tissue fibrosis, and CD31 (Abcam ab182981) was used to evaluate myocardial vessels, which were subsequently examined by a pathologist who was blinded to the ultrasonic results.

### 2.6. Statistical analysis

The Shapiro–Wilk test was performed to evaluate the distribution of the data. Continuous and normally distributed data were presented as mean ± SD. Differences among groups were determined by one-way ANOVA followed by Bonferroni *post hoc* tests. A value of *p* < 0.05 was considered significant. All statistical analyses were performed by SPSS (version 21.0, SPSS Inc., Chicago, IL, USA), and GraphPad Prism (version 7.04, GraphPad Software, Inc., La Jolla, CA, USA) was used to generate statistical figures.

## 3. Results

### 3.1. Comparison of conventional echo-Doppler results

**Figure 2:**
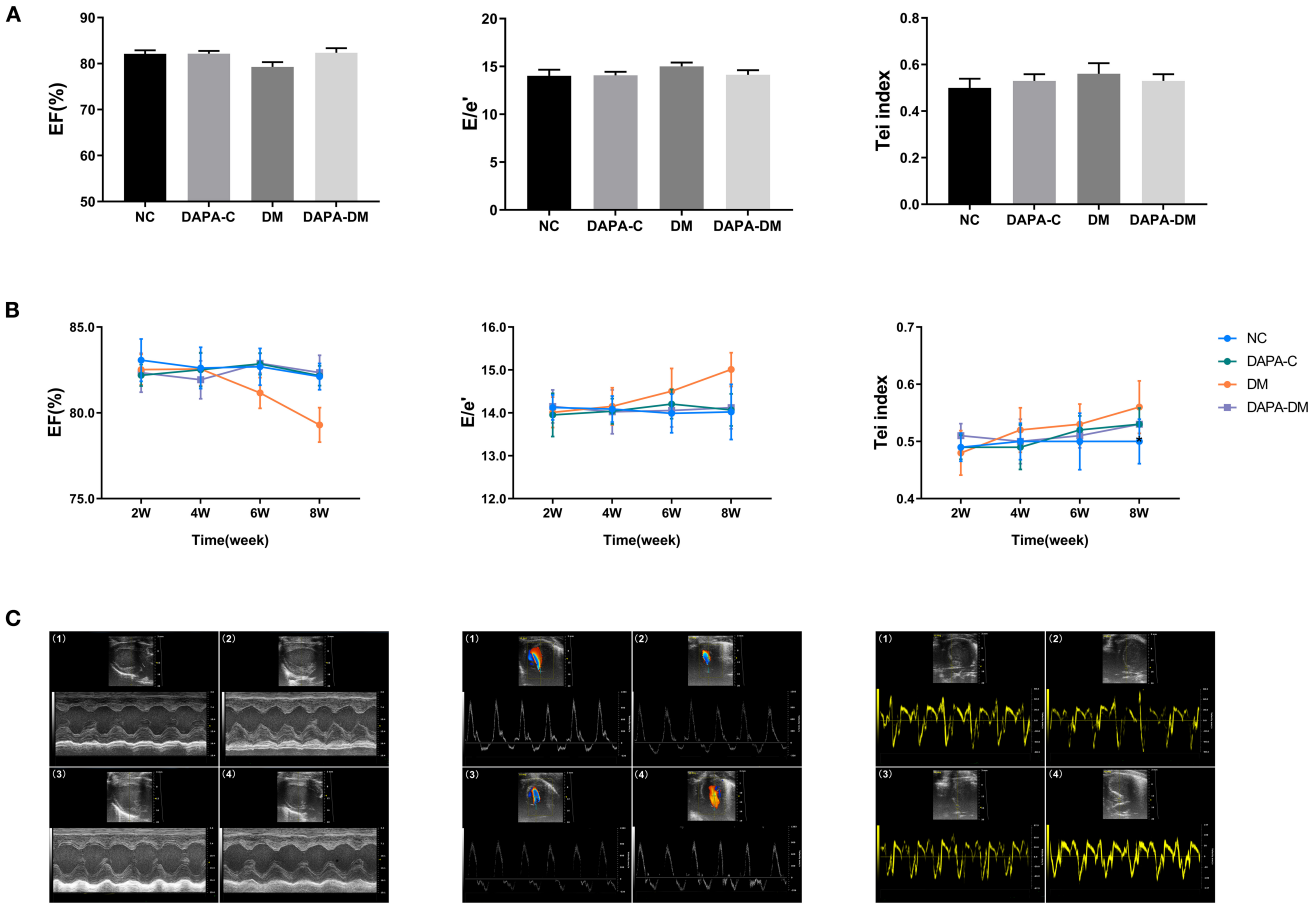
Comparison of conventional echo-Doppler data among groups. **(A)** EF, E/e', and Tei of the four groups at different time points. **(B)** Differences in EF, E/e', and Tei among the four groups at different time points. **(C)** Representative M-mode images, spectral Doppler images, and tissue Doppler images of the four groups at 8 weeks. (1) normal control group, (2) DAPA-control group, (3) diabetic group, (4) DAPA-diabetic group. EF, ejection fraction; E, peak flow velocity; e', myocardial motion velocity; Tei, Tei index; NC, normal control group; DAPA-C, DAPA-control group; DM, diabetic group; DAPA-DM, DAPA-diabetic group.

Left ventricular geometric measurements, E/e' and Tei index were unchanged among the four groups from 2 to 8 weeks (all *p* > 0.05). The left ventricular EF decreased gradually in the diabetic group but remained stable in the DAPA-diabetic group. However, the difference did not reach statistical significance (Table 1, Figure 2).

**Table 1:** Conventional echo-Doppler measurements.

	**Normal control**				**DAPA-control**				**Diabetic**				**DAPA-diabetic**			

	**2W (*****n** =* **8)**	**4W (*****n** =* **8)**	**6W (*****n** =* **8)**	**8W (*****n** =* **8)**	**2W (*****n** =* **8)**	**4W (*****n** =* **8)**	**6W (*****n** =* **8)**	**8W (*****n** =* **8)**	**2W (*****n** =* **8)**	**4W (*****n** =* **8)**	**6W (*****n** =* **8)**	**8W (*****n** =* **8)**	**2W (*****n** =* **8)**	**4W (*****n** =* **8)**	**6W (*****n** =* **8)**	**8W (*****n** =* **8)**
IVSd (mm)	1.81 ± 0.21	1.80 ± 0.21	1.77 ± 0.09	1.91 ± 0.13	1.75 ± 0.15	1.82 ± 0.14	1.85 ± 0.13	1.86 ± 0.10	1.78 ± 0.20	1.81 ± 0.12	1.87 ± 0.12	1.97 ± 0.17	1.84 ± 0.12	1.79 ± 0.16	1.82 ± 0.14	1.89 ± 0.12
EDD (mm)	6.92 ± 0.25	7.05 ± 0.32	7.20 ± 0.17	7.19 ± 0.26	6.88 ± 0.46	7.13 ± 0.25	7.17 ± 0.14	7.24 ± 0.32	6.82 ± 0.37	7.20 ± 0.28	7.02 ± 0.44	6.98 ± 0.26	6.82 ± 0.38	7.05 ± 0.39	7.28 ± 0.32	7.24 ± 0.49
ESD (mm)	3.09 ± 0.44	3.13 ± 0.42	3.38 ± 0.27	3.43 ± 0.19	3.21 ± 0.17	3.36 ± 0.21	3.36 ± 0.17	3.45 ± 0.17	3.18 ± 0.32	3.39 ± 0.18	3.43 ± 0.33	3.55 ± 0.20	3.33 ± 0.24	3.36 ± 0.18	3.41 ± 0.21	3.47 ± 0.18
PWd (mm)	1.86 ± 0.14	1.81 ± 0.24	1.88 ± 0.15	1.92 ± 0.09	1.80 ± 0.20	1.81 ± 0.17	1.87 ± 0.07	1.96 ± 0.10	1.88 ± 0.19	1.86 ± 0.12	1.94 ± 0.22	1.98 ± 0.16	1.82 ± 0.17	1.87 ± 0.15	1.87 ± 0.20	1.83 ± 0.16
EF (%)	83.07 ± 3.49	82.62 ± 3.41	82.69 ± 3.03	82.11 ± 2.17	82.19 ± 1.77	82.51 ± 2.75	82.85 ± 1.77	82.16 ± 1.64	82.52 ± 2.51	82.55 ± 2.72	81.16 ± 2.53	79.30 ± 2.83	82.34 ± 3.21	81.93 ± 3.13	82.89 ± 1.63	82.35 ± 2.84
FS (%)	53.36 ± 4.17	52.97 ± 3.89	53.05 ± 3.34	52.34 ± 2.48	52.23 ± 1.98	52.79 ± 3.01	53.13 ± 1.99	52.38 ± 1.85	52.75 ± 2.80	52.86 ± 3.06	51.23 ± 2.74	49.42 ± 2.64	51.13 ± 3.69	52.13 ± 3.47	53.21 ± 1.81	52.52 ± 3.40
LVMI (mg/g)	3.29 ± 0.41	2.10 ± 0.28	1.82 ± 0.22	1.76 ± 0.22	3.35 ± 0.59	2.09 ± 0.18	1.88 ± 0.34	1.90 ± 0.31	3.24 ± 0.21	1.77 ± 0.17	1.57 ± 0.13	1.49 ± 0.31	3.39 ± 0.50	1.83 ± 0.27	1.73 ± 0.14	1.67 ± 0.31
EDV (uL)	244.87 ± 33.99	268.12 ± 26.33	272.58 ± 14.77	271.85 ± 22.31	242.85 ± 32.43	266.79 ± 20.94	269.60 ± 47.01	276.35 ± 28.57	241.50 ± 29.57	272.28 ± 23.80	262.77 ± 40.61	254.48 ± 21.85	241.79 ± 30.78	260.05 ± 32.02	276.98 ± 31.43	279.21 ± 36.88
ESV (uL)	40.05 ± 10.63	46.37 ± 8.77	47.29 ± 9.43	48.55 ± 6.42	43.22 ± 4.02	46.50 ± 6.97	46.27 ± 5.48	49.21 ± 5.94	40.81 ± 10.10	47.20 ± 6.08	48.94 ± 10.69	52.78 ± 7.35	43.10 ± 4.72	46.44 ± 5.92	47.88 ± 7.17	50.02 ± 6.47
E/e'	14.13 ± 0.83	14.09 ± 0.86	13.99 ± 1.28	14.02 ± 1.82	13.95 ± 1.42	14.04 ± 0.84	14.21 ± 0.96	14.07 ± 1.06	14.01 ± 1.01	14.15 ± 1.23	14.50 ± 1.50	15.01 ± 1.11	14.15 ± 1.09	14.02 ± 1.45	14.06 ± 1.09	14.12 ± 1.39
Tei index	0.49 ± 0.07	0.50 ± 0.09	0.50 ± 0.14	0.50 ± 0.11	0.49 ± 0.06	0.49 ± 0.11	0.52 ± 0.07	0.53 ± 0.08	0.48 ± 0.11	0.52 ± 0.11	0.53 ± 0.10	0.56 ± 0.13	0.51 ± 0.06	0.50 ± 0.11	0.51 ± 0.06	0.53 ± 0.08

IVSd, interventricular septum thickness in diastole; EDD, Left ventricular end-diastolic diameter; ESD, Left ventricular end-systolic diameter; PWd, Left ventricular end-diastolic posterior wall thickness; EF, ejection fraction; FS, fractional shortening; LVM, Left ventricular mass; LVMI, Left ventricular mass index; EDV, Left ventricular end-diastolic volume; ESV, Left ventricular end-systolic volume; E, peak flow velocity; e', myocardial motion velocity.

### 3.2. Comparison of 2D-STE data

In the diabetic group, GLS decreased stepwise from 2 to 8 weeks, with a statistically significant difference between 2 and 8 weeks (*p* = 0.02). At 8 weeks, left ventricular strain parameters were significantly reduced in the diabetic group compared to the normal control (*p* = 0.011) and DAPA-control groups (*p* = 0.032). However, no differences were observed among the normal control, DAPA-control, and DAPA-diabetic groups (Table 2, Figure 3).

**Table 2:** Left ventricular strain data by 2D-STE.

		**Normal control (*n =* 32)**	**DAPA-control (*n =* 32)**	**Diabetic (*n =* 32)**	**DAPA-diabetic (*n =* 32)**
2W	GLS (%)	−21.78 ± 2.13	−20.04 ± 3.21	−20.95 ± 3.14	−20.34 ± 3.52
	GCS (%)	−20.23 ± 2.31	−20.92 ± 3.05	−21.59 ± 3.21	−20.26 ± 3.86
	GRS (%)	47.82 ± 2.55	46.95 ± 6.58	48.41 ± 5.53	46.49 ± 4.53
4W	GLS (%)	−21.31 ± 2.18	−19.13 ± 3.66	−19.08 ± 2.75	−19.75 ± 3.02
	GCS (%)	−21.68 ± 2.66	−21.10 ± 2.29	−21.09 ± 2.63	−21.80 ± 4.62
	GRS (%)	48.01 ± 3.15	46.71 ± 5.20	46.21 ± 6.10	47.24 ± 3.82
6W	GLS (%)	−20.93 ± 3.12	−19.03 ± 2.24	−18.98 ± 3.35	−18.45 ± 3.68
	GCS (%)	−20.19 ± 2.97	−19.62 ± 2.03	−19.14 ± 2.35	−19.88 ± 3.43
	GRS (%)	48.41 ± 4.81	47.76 ± 4.91	45.12 ± 5.24	46.81 ± 5.83
8W	GLS (%)	−21.02 ± 2.96	−20.42 ± 3.43	−16.24 ± 2.42^*^^#^^†^	−19.09 ± 2.06
	GCS (%)	−20.58 ± 4.07	−19.44 ± 3.62	−18.24 ± 2.23	−19.18 ± 2.49
	GRS (%)	46.83 ± 3.75	46.55 ± 5.71	43.55 ± 4.52	46.14 ± 5.25

GLS, global peak longitudinal strain; GRS, global peak radial strain; GCS, global peak circumferential strain.

* *p* < 0.05 vs. normal control group;

† *p* < 0.05 vs. DAPA-control group;

# *p* < 0.05 vs. diabetic group at 2 weeks.

**Figure 3:**
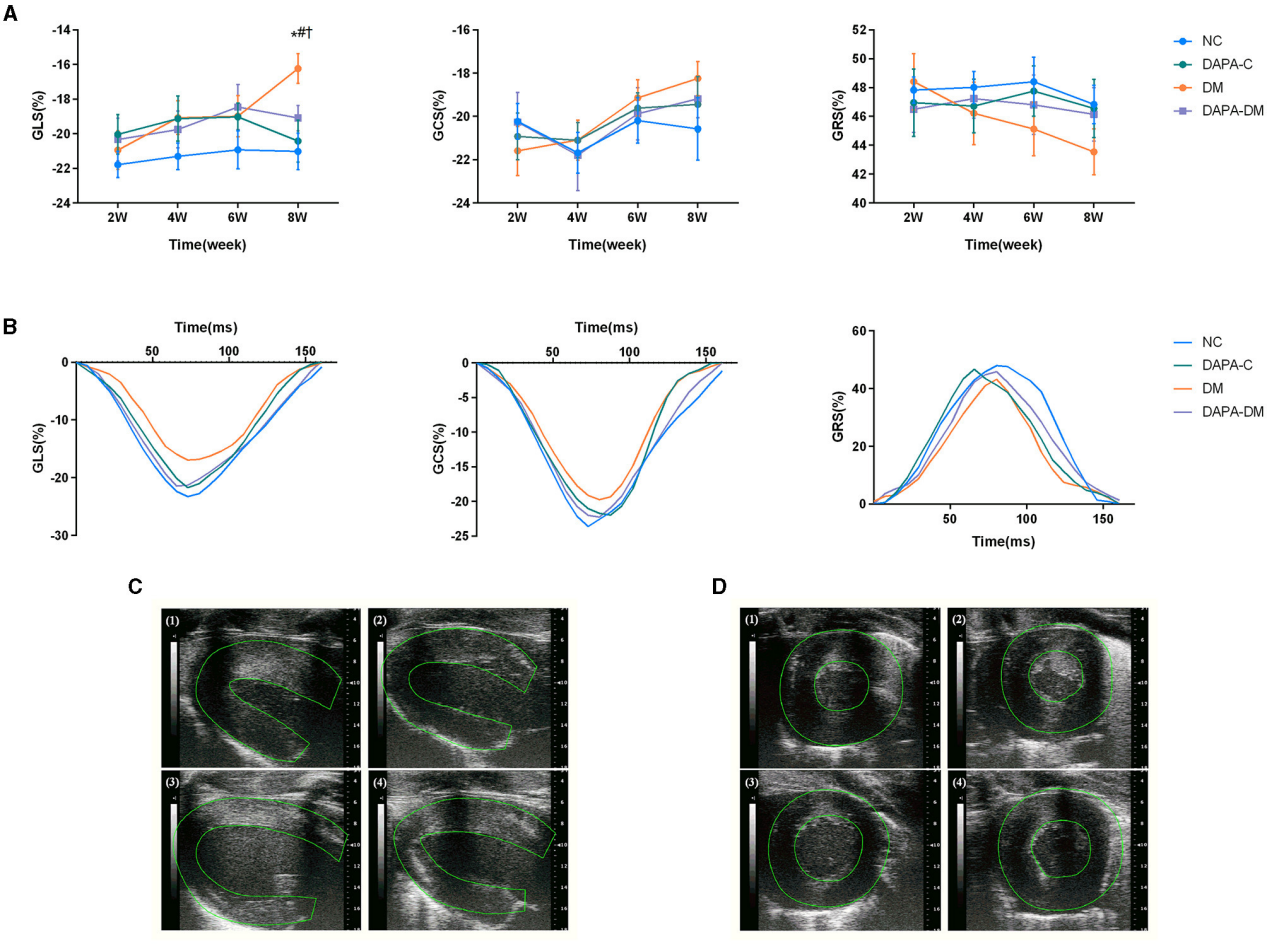
Myocardial strain assessed by 2D-STE. **(A)** GLS, GRS and GCS scores of the four groups at different time points. **(B)** Representative strain time curves of the four groups. **(C)** Representative long-axis strain images of the four groups at 8 weeks. **(D)** Representative short-axis strain images of the four groups at 8 weeks. (1) normal control group, (2) DAPA-control group, (3) diabetic group, (4) DAPA-diabetic group. GLS, global peak longitudinal strain; GRS, global peak radial strain; GCS, global peak circumferential strain; NC, normal control group; DAPA-C, DAPA-control group; DM, diabetic group; DAPA-DM, DAPA-diabetic group. *p < 0.05 vs. normal control group; ^†^*p* < 0.05 vs. DAPA-control group; ^#^*p* < 0.05 vs. diabetic group at 2 weeks.

### 3.3. Comparison of MCE quantitative data

In the diabetic group, the values of myocardial perfusion as expressed by MBFV and MBF decreased stepwise from 2 to 8 weeks, with statistically significant differences between 2 and 6 weeks (*p* = 0.019, *p* = 0.023) and 2 and 8 weeks (*p* = 0.001, *p* = 0.001). Compared with the normal control and DAPA-control groups, MBFV, MBF, and MBV were decreased gradually in the diabetic group across 2, 4, 6, and 8 weeks, reaching levels of statistically significant differences at 6 weeks for MBFV and MBF (*p* = 0.047, *p* = 0.026) and at 8 weeks for MBV (*p* = 0.033). There were no significant differences in these values among the normal control, DAPA-control, and DAPA-diabetic groups (Table 3, Figure 4).

**Table 3:** Changes in myocardial perfusion parameters by MCE.

		**Normal control (*n =* 32)**	**DAPA-control (*n =* 32)**	**Diabetic (*n =* 32)**	**DAPA-diabetic (*n =* 32)**
2W	MBV (dB)	11.82 ± 1.94	10.94 ± 1.70	11.01 ± 2.33	10.75 ± 2.33
	MBFV (/s)	10.83 ± 1.17	10.91 ± 2.89	11.35 ± 1.96	11.08 ± 2.41
	MBF (dB/s)	128.59 ± 29.50	118.82 ± 35.95	123.04 ± 22.59	119.27 ± 38.62
4W	MBV (dB)	11.70 ± 1.37	11.19 ± 1.65	10.23 ± 1.08	11.09 ± 1.06
	MBFV (/s)	11.11 ± 2.14	10.99 ± 2.40	10.01 ± 1.98	10.39 ± 2.46
	MBF (dB/s)	128.58 ± 22.43	124.48 ± 39.40	103.41 ± 27.44	114.45 ± 26.18
6W	MBV (dB)	11.63 ± 2.82	11.33 ± 1.60	10.13 ± 1.88	10.66 ± 2.16
	MBFV (/s)	11.27 ± 1.94	10.42 ± 2.64	8.70 ± 0.83^*^^#^	9.44 ± 1.20
	MBF (dB/s)	129.03 ± 31.38	116.91 ± 28.46	88.04 ± 18.39^*^^#^	100.85 ± 25.51
8W	MBV (dB)	11.90 ± 1.91	11.11 ± 1.42	9.51 ± 1.53^*^	10.58 ± 1.43
	MBFV (/s)	10.99 ± 2.05	10.57 ± 1.58	7.86 ± 1.51^**^^##^^†^	9.58 ± 1.68
	MBF (dB/s)	130.96 ± 33.11	117.41 ± 24.84	74.85 ± 19.22^**^^##^^†^	102.13 ± 24.28

MBV, myocardial blood volume; MBFV, myocardial blood flow velocity; MBF, myocardial blood flow.

* *p* < 0.05,

** *p* < 0.01 vs. normal control group;

† *p* < 0.05 vs. DAPA-control group;

# *p* < 0.05,

## *p* < 0.01 vs. diabetic group at 2 weeks.

**Figure 4:**
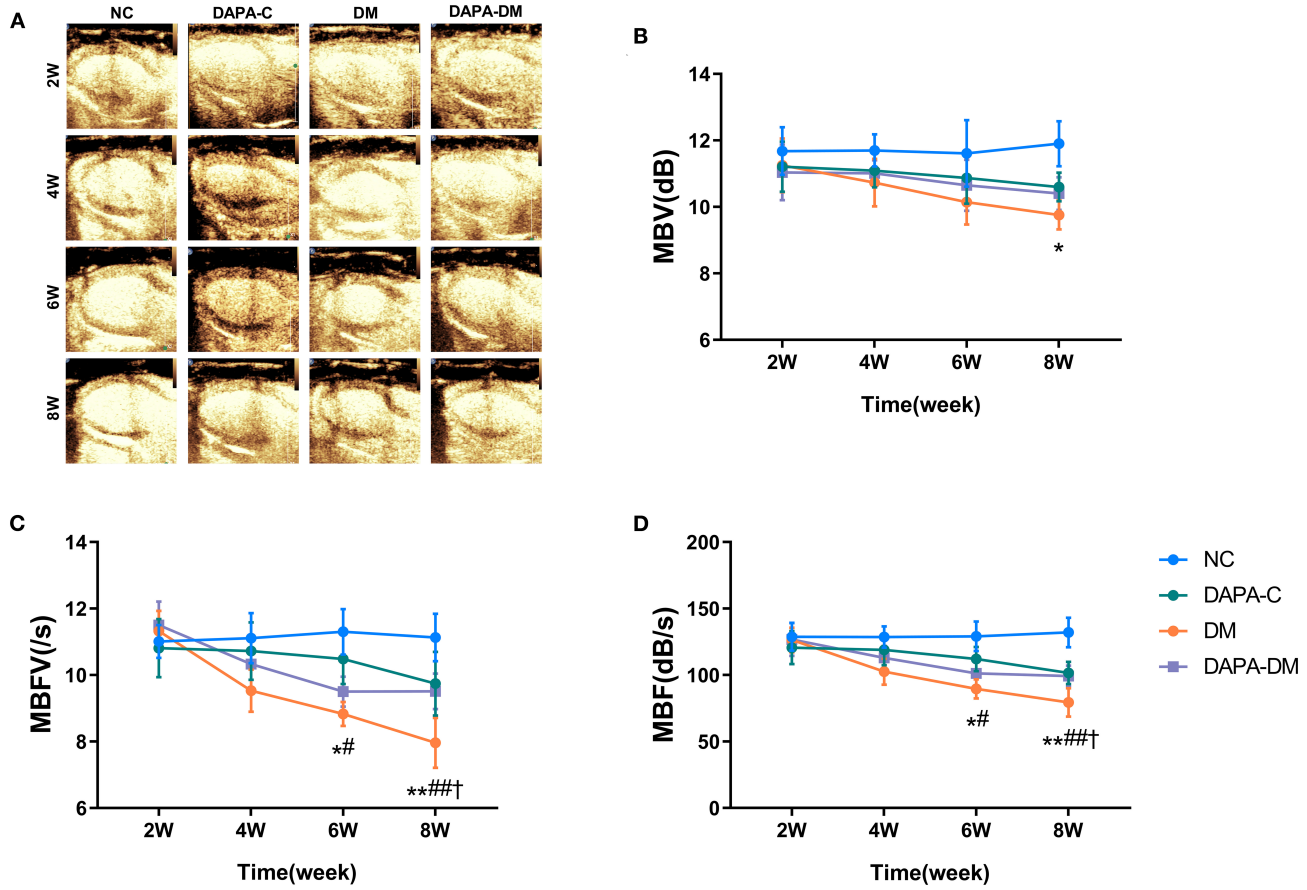
Myocardial perfusion assessed by MCE. **(A)** Representative MCE-based myocardial perfusion images of the four groups at different time points. **(B)** Differences in the MBV among the four groups at different time points. **(C)** Differences in MBFV among the four groups at different time points. **(D)** Differences in MBF among the four groups at different time points. MBV, myocardial blood volume; MBFV, myocardial blood flow velocity; MBF, myocardial blood flow; NC, normal control group; DAPA-C, DAPA-control group; DM, diabetic group; DAPA-DM, DAPA-diabetic group. *p < 0.05, **p < 0.01 vs. normal control group; ^†^*p* < 0.05 vs. DAPA-control group; ^#^*p* < 0.05, ^##^*p* < 0.01 vs. diabetic group at 2 weeks.

### 3.4. Histopathological findings

**Figure 5:**
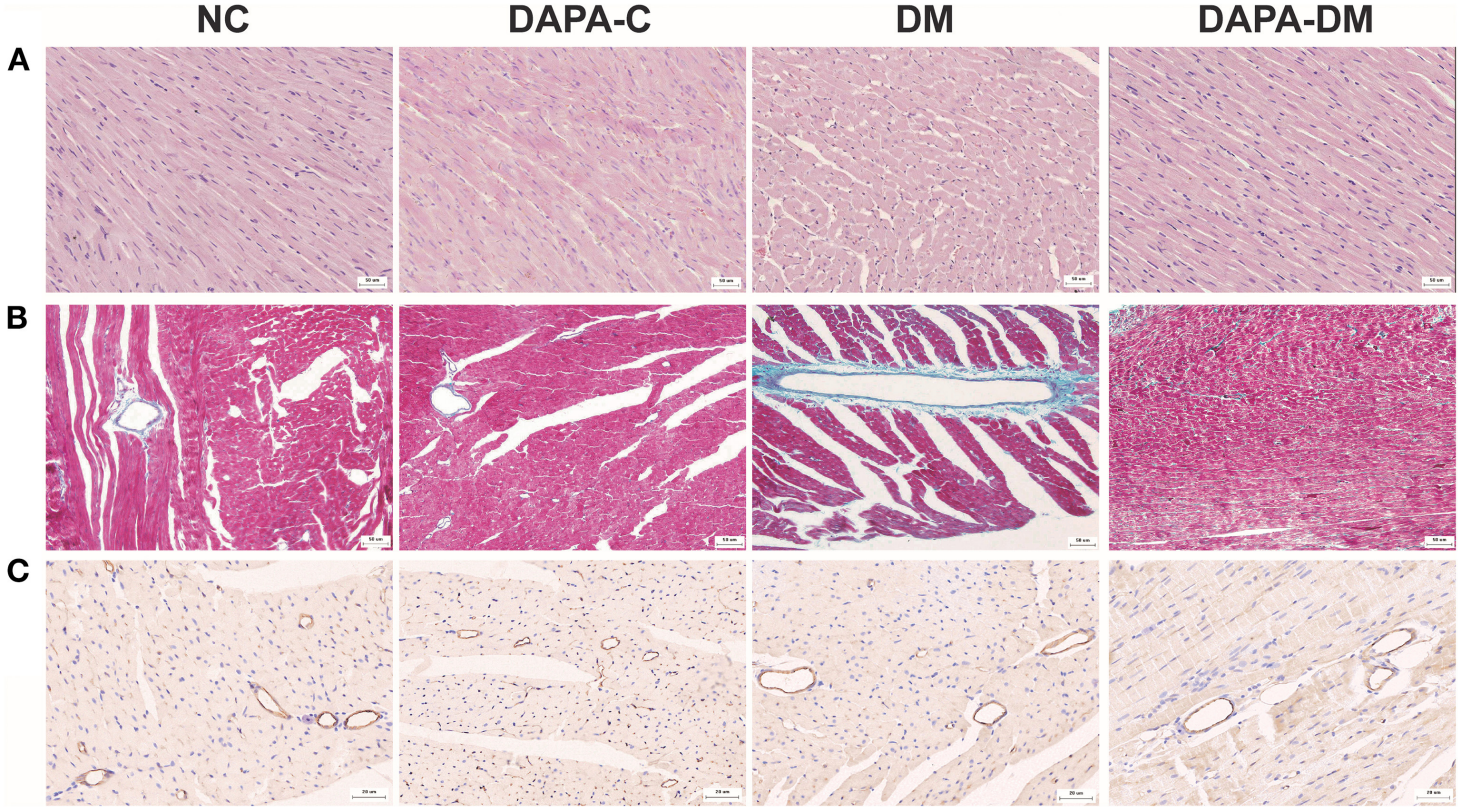
Typical pathological images of the four groups at 8 weeks. **(A)** Representative H&E staining images of the four groups at 8 weeks. Magnification: × 200. **(B)** Representative Masson's trichrome staining images of the four groups at 8 weeks. Magnification: × 200. **(C)** Representative CD31 staining images of the four groups at 8 weeks. Magnification: × 400. NC, normal control group; DAPA-C, DAPA-control group; DM, diabetic group; DAPA-DM, DAPA-diabetic group.

Histopathological changes were found in the diabetic group. Figure 5 shows typical tissue morphology following H&E, Masson's trichrome, and CD31 staining in each group at 8 weeks. In the normal control group, myocardial cell morphology was normal, with closely packed cells. In contrast, in the diabetic group, myocardial cells were vacuolated and disorganized; increased collagen fibers deposition and decreased myocardial microvascular density were also seen. However, in the DAPA-diabetic group, the morphology of myocardial cells was essentially normal, with closely packed cells. In addition, myocardial fibrosis and myocardial microvascular density decreased were not as obvious.

## 4. Discussion

The results of this study showed that the combined use of 2D-STE and MCE was a sensitive way to detect left ventricular deformity and impaired microvascular perfusion in prediabetes and earlyDM. DAPA exerts a cardio-protective effect in this setting.

Myocardial dysfunction of varying degrees often occurs in earlyDM (15). With the prolongation of the disease process, disturbance of energy metabolism causes myocardial and endothelial cell remodeling, apoptosis, fibrosis and myocardial microvascular abnormalities (16–18). In addition, increased wall thickness and mass reduce left ventricular diastolic compliance (19). Eventually, left ventricular systolic function declines, leading to diabetic cardiomyopathy. Therefore, timely detection of early changes in cardiac structure and function in diabetic patients is of great clinical relevance (20). In this study, we built a diabetic model that was first fed a HFD for 8 weeks to induce insulin resistance and then injected with STZ to induce high glucose (21–23). Next, we performed comprehensive echo assessments at different time points in the process from prediabetes to earlyDM to evaluate the dynamic evolution of myocardial function and microvascular perfusion.

One major finding of this study is the advantage of 2D-STE and MCE, in comparison with conventional echo-Doppler techniques, for early detection of left ventricular deformity and abnormal microvascular and myocardial perfusion in prediabetes and earlyDM. In fact, no significant changes in left ventricular geometry or systolic and diastolic function were observed using conventional echo-Doppler parameters in diabetic rats, which is consistent with previous studies (24). Although tissue Doppler imaging has been utilized to evaluate myocardial motion, especially when its direction is parallel to sound velocity, the accuracy of such measurements is angle dependent, limiting its value in assessing cardiac function in patients with diabetes (25). 2D-STE regards the myocardium as uniformly distributed acoustic spots and then tracks each spot and calculates its motion trajectory to accurately determine myocardial strain and strain rate (3). Previous studies have shown that 2D-STE can effectively reveal minor myocardial damage and provide quantitative assessment of left ventricular function in coronary heart disease (26), diabetic cardiomyopathy (27), and structural heart disease (28). Importantly, GLS is helpful in the early detection of subclinical myocardial dysfunction with preserved left ventricular EF for diabetic patients (27, 29). Likewise, MCE is adopted to dynamically evaluate the functional changes in myocardial microcirculation. Signal intensity in MCE imaging (microbubble volume) is thought to be equivalent to corresponding myocardial blood flow and has been used to estimate the local status of myocardial perfusion. Additionally, MCE has excellent temporal and spatial resolution and is low cost and simple to operate. Numerous experiments have shown that MCE is an effective method for detecting abnormal myocardial blood flow perfusion (4) and that it is superior to positron emission tomography (PET) and single photon emission computed tomography (SPECT) (30).

Another important finding is that DAPA exerts a beneficial effect on left ventricular function and myocardial perfusion in diabetic rats. SGLT2 inhibitors are a new class of oral hypoglycemic drugs that can lower blood glucose levels by inhibiting sodium-glucose reabsorption in proximal renal tubules and by promoting urine glucose excretion. These agents not only effectively reduce glycosylated hemoglobin but also have a low risk of hypoglycemia. Recent studies have confirmed that DAPA slightly decreases systolic and diastolic blood pressure, improves myocardial energy utilization, and inhibits myocardial fibrosis. Recently, DAPA has been embraced as a drug of choice for the treatment of heart failure with reduced or preserved EF in diabetic and non-diabetic patients (5, 10). In view of the significant advantages of DAPA for cardiovascular benefits, we intended to apply DAPA in prediabetes and earlyDM to explore its role in reversing myocardial dysfunction by improving myocardial strain and microcirculation in a rat model. Our results of 2D-STE and MCE studies showed that indexes of left ventricular deformity (GLS, GCS, and GRS) and parameters of myocardial perfusion (MBFV, MBF, and MBV) gradually recovered to baseline levels after treatment with DAPA, suggesting that the SGLT2 inhibitor DAPA may have cardioprotective effects in this diabetic setting.

In addition, our histopathological study demonstrated that myocardial cells were vacuolated and disorganized in prediabetes and earlyDM. Increased collagen fibers deposition and decreased myocardial microvascular density were also seen, which is in line with previous reports (31). Interestingly, after treatment with DAPA, myocardial cells became essentially normal with closely packed cells. Furthermore, myocardial fibrosis and myocardial microvascular density decreased were not as obvious. These results further proved the effect of cardiovascular protection caused by DAPA.

In conclusion, the findings of this experimental study support the view that the combined use of 2D-STE and MCE is more sensitive than the conventional Doppler-echo technique for detecting impaired myocardial function and microvascular perfusion in prediabetes and earlyDM. DAPA exerts a beneficial effect on protecting left ventricular function and myocardial perfusion in diabetic rats. This information may provide experimental basis for the pathophysiology of diabetic cardiomyopathy and should be useful to physicians who are involved in decision-making for diabetic patients.

This study is subject to several limitations. First, the current study focused on the changes in left ventricular strain and microvascular perfusion in prediabetes and the early stage of diabetes mellitus and the effect of DAPA but did not focus on the changes in the late stage and the corresponding role of DAPA. Future studies on the changes in the late stage of diabetes and the role of DAPA will be carried out. Second, in the present study, resting MCE was performed; however, in the future, stress MCE may be used to further evaluate myocardial microvascular perfusion.

## Data availability statement

The original contributions presented in the study are included in the article/Supplementary material, further inquiries can be directed to the corresponding authors.

## Ethics statement

The animal study was reviewed and approved by Laboratory Animal Welfare and Ethics Committee of the Third Military Medical University.

## Author contributions

JL, YW, YSha, and YShe participated in study design, data analysis and interpretation, and drafting the manuscript. JL, JZ, XL, and LT performed experiments. JL, HH, YD, YSha, and YShe revised the manuscript before final approval. All authors read and approved the final manuscript.
