# Corrigendum: Dynamic evolution of left ventricular strain and microvascular perfusion assessed by speckle tracking echocardiography and myocardial contrast echocardiography in diabetic rats: effect of dapagliflozin

**DOI:** 10.3389/fcvm.2024.1452088

**Published:** 2024-07-09

**Authors:** Juan Liu, Yixuan Wang, Jun Zhang, Xin Li, Lin Tan, Haiyun Huang, Yang Dai, Yongning Shang, Ying Shen

**Affiliations:** ^1^ Department of Ultrasound, Southwest Hospital, Army Medical University (Third Military Medical University), Chongqing, China; ^2^ Department of Cardiovascular Medicine, Rui Jin Hospital, Shanghai Jiaotong University School of Medicine, Shanghai, China

**Keywords:** early diabetes mellitus, microvascular strain, microvascular perfusion, speckle tracking echocardiography, myocardial contrast echocardiography, dapagliflozin

**A Corrigendum on**
Dynamic evolution of left ventricular strain and microvascular perfusion assessed by speckle tracking echocardiography and myocardial contrast echocardiography in diabetic rats: effect of dapagliflozin By Liu J, Wang Y, Zhang J, Li X, Tan L, Huang H, Dai Y, Shang Y, Shen Y (2023). Front. Cardiovasc. Med. 10:1109946. doi: 10.3389/fcvm.2023.1109946

In the published article, there was an error in Figure 3C as published. Figure 3C is a sampling diagram of the left ventricular global longitudinal strain (GLS) analysis of the four groups [(1) normal control group; (2) DAPA-control group; (3) diabetic group; (4) DAPA-diabetic group] at 8 weeks. In the process of combining the original figure (1)–(4) into Figure 3C, parts (3) and (4) were accidentally replaced with part(2). The corrected Figure 3C and its caption appear below.

**Figure 3:**
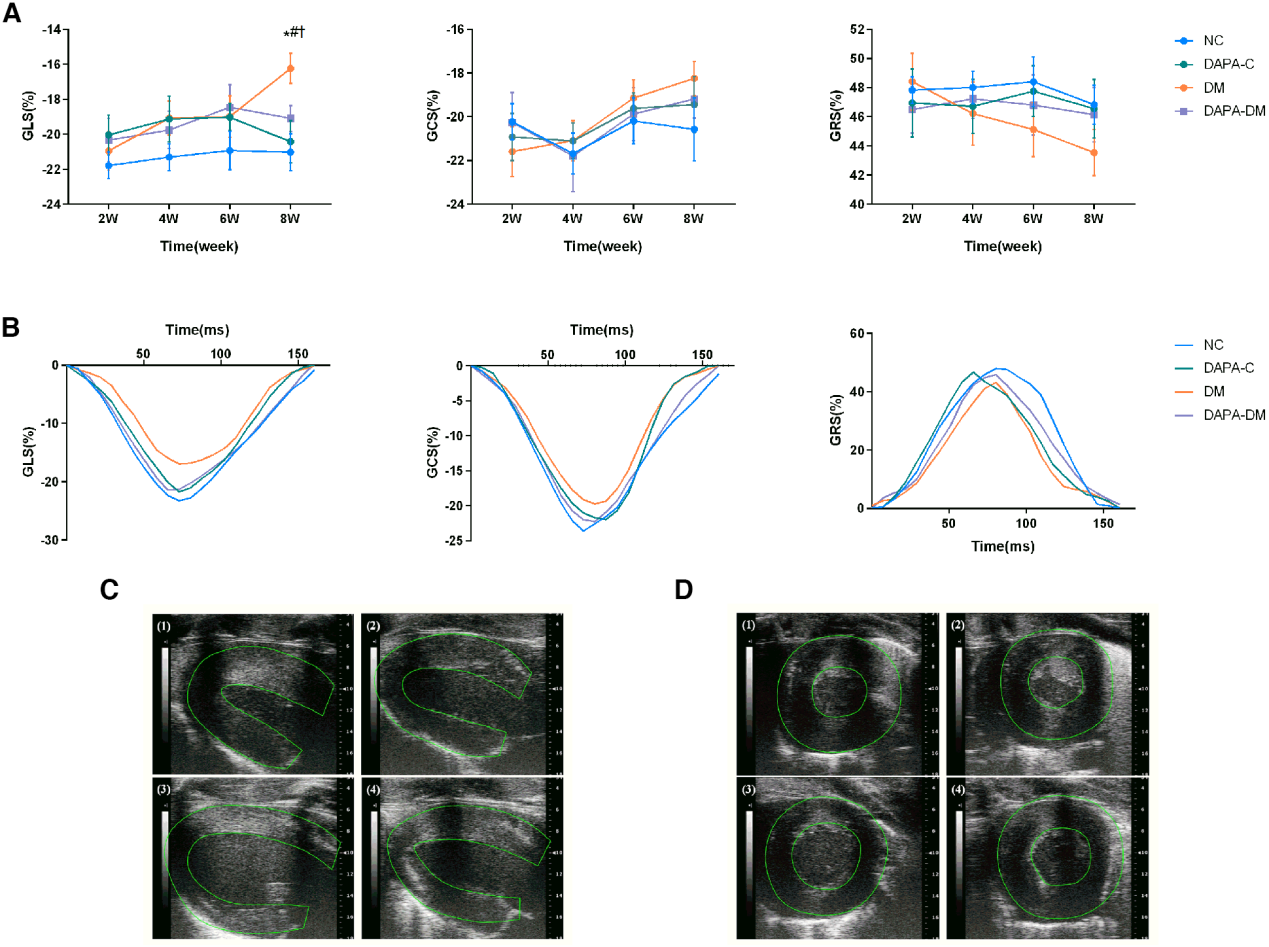
Myocardial strain assessed by 2D-STE. (**A**) GLS, GRS and GCS scores of the four groups at different time points. (**B**) Representative strain time curves of the four groups. (**C**) Representative long-axis strain images of the four groups at 8 weeks. (**D**) Representative short-axis strain images of the four groups at 8 weeks. (1) Normal control group, (2) DAPA-control group, (3) diabetic group, (4) DAPA-diabetic group. GLS, global peak longitudinal strain; GRS, global peak radial strain; GCS, global peak circumferential strain. NC, normal control group; DAPA-C, DAPA-control group; DM, diabetic group; DAPA-DM, DAPA-diabetic group. *p < 0.05 vs. normal control group; ^†^*p *< 0.05 vs. DAPA-control group; ^#^*p *< 0.05 vs. diabetic group at 2 weeks.

The authors apologize for this error and state that this does not change the scientific conclusions of the article in any way. The original article has been updated.

## Publisher's note

All claims expressed in this article are solely those of the authors and do not necessarily represent those of their affiliated organizations, or those of the publisher, the editors and the reviewers. Any product that may be evaluated in this article, or claim that may be made by its manufacturer, is not guaranteed or endorsed by the publisher.

