# Supplementary material for: Dynamic evolution of left ventricular strain and microvascular perfusion assessed by speckle tracking echocardiography and myocardial contrast echocardiography in diabetic rats: Effect of dapagliflozin
Source: Front Cardiovasc Med. 2023 Feb 23;10:1109946. doi: 10.3389/fcvm.2023.1109946 (PMC9996187; doi:10.3389/fcvm.2023.1109946)
Supplement: Supplementary file 1 [file Table_1.DOCX]

| **Table 1** Body weight of the animals. | | | | |
| --- | --- | --- | --- | --- |
|  | Normal control  (*n* = 32) | DAPA-control  (*n* = 32) | Diabetic  (*n* = 32) | DAPA-diabetic  (*n* = 32) |
| 2W | 214.00±10.18 | 210.63±16.02 | 216.38±18.58 | 207.75±6.56 |
| 4W | 356.38±23.66 | 355.50±30.33 | 434.50±26.47 | 406.13±33.42 |
| 6W | 431.00±34.94 | 416.75±16.87 | 495.63±37.28 | 455.75±54.97 |
| 8W | 462.88±28.24 | 433.75±32.62 | 555.75±40.70 | 495.63±66.20* |
| ^*^ *p* < 0.05 vs. diabetic group. | | | | |
